# Optimizing prone CT use for suspected interstitial lung abnormalities

**DOI:** 10.1007/s00330-024-11259-5

**Published:** 2024-12-18

**Authors:** Jiyoung Song, Kum Ju Chae, Jong Eun Lee, Masahiro Yanagawa, Jonathan H. Chung, David A. Lynch, Myoung-Jin Jang, Jin Mo Goo, Soon Ho Yoon

**Affiliations:** 1 Department of Radiology, Seoul National University Hospital, Seoul National College of Medicine, Seoul, Korea; 2 Department of Radiology, Jeonbuk National University Hospital, Jeonju, Korea; 3 Department of Radiology and Research Institute of Radiology, Asan Medical Center, Seoul, Korea; 4 Department of Radiology, Osaka University Graduate School of Medicine, Osaka, Japan; 5 Department of Radiology, University of California San Diego, San Diego, USA; 6 Department of Radiology, National Jewish Health, Denver, USA; 7 Medical Research Collaborating Center, Seoul National University Hospital, Seoul, Republic of Korea

**Keywords:** Lung diseases (Interstitial), Pulmonary atelectasis, Tomography (X-ray computed), Interstitial lung abnormality, Prone CT

## Abstract

**Objectives:**

We investigated whether supine chest CT alone suffices for diagnosing ILAs, thereby reducing the need for prone chest CT.

**Materials and methods:**

Patients who underwent prone chest CT for suspected ILAs from January 2021 to July 2023, with matching supine CT within 1 year, were retrospectively evaluated. Five multinational thoracic radiologists independently rated ILA suspicion and fibrosis scores (1 to 5-point) and ILA extent (1–100%) using supine CT first, then combined supine-prone CT after a 1-month washout. We categorized ILA suspicion and fibrosis scores into four diagnostic groups; normal, non-fibrotic, indeterminate-type, and fibrotic ILAs. The areas under the receiver operating characteristic curve (AUCs) of ILA suspicion scores, inter-reader agreement on diagnostic categories, and intra-reader/inter-reader reliability for ILA extent were evaluated.

**Results:**

This study included 69 patients (mean age 67.2 ± 7.2 years; 36 women), with 23 age- and sex-matched patients in each group: normal, non-fibrotic ILAs, and fibrotic ILAs. The pooled AUC for ILA suspicion and inter-reader agreement on diagnostic categories improved for non-fibrotic ILAs with prone CT (AUC 0.76 to 0.92, *p* < 0.001; Fleiss kappa 0.25 to 0.51, *p* = 0.004), but not for fibrotic ILAs (AUC 0.94 to 0.99, *p* = 0.06; Fleiss kappa 0.63 to 0.72, *p* = 0.08). ILA extent was 1–2% smaller with prone CT for both ILA types (*p* < 0.001).

**Conclusion:**

For fibrotic ILAs, supine CT alone exhibited substantial diagnostic accuracy and inter-reader agreement, while the diagnosis of non-fibrotic ILAs benefited from adding prone CT. Supine CT alone slightly overestimated extent regardless of ILA type.

**Key Points:**

***Question*** *Prone CT is recommended when interstitial lung abnormalities (ILAs) are suspected on supine CT, but its benefits remain underexplored.*

***Findings*** *Supine CT alone sufficed for diagnosing fibrotic ILAs, while prone CT improved non-fibrotic ILA diagnosis and reduced extent overestimation for both types.*

***Clinical relevance*** *Omitting prone CT reduces extra time, space, and radiation exposure without compromising the diagnosis of fibrotic ILAs, which have higher rates of progression and mortality risks, enhancing patient comfort and simplifying patient management.*

## Introduction

Interstitial lung abnormalities (ILAs) are incidental CT findings that suggest early morphological changes of interstitial lung disease (ILD) but are detected without clinical suspicion of ILD [1, 2]. These findings include ground-glass opacities, reticular opacities, architectural distortion, traction bronchiectasis/bronchiolectasis, honeycombing, and non-emphysematous cysts [2, 3]. ILAs are further subcategorized as fibrotic or non-fibrotic, with fibrosis manifesting as traction bronchiectasis, architectural distortion, and honeycombing [1–4]. Subpleural lower-lobe predominance and traction bronchiectasis are related to an elevated risk of progression [5].

The reported prevalence of ILAs ranges from 4 to 9% in smokers and 2 to 7% in non-smokers [2], with a pooled prevalence of 7% in both lung cancer screening and the general population [6]. The incidence of ILAs is estimated to be 13.1 cases per 1000 person-years [7]. Although ILAs are often asymptomatic, they are correlated with unfavorable clinical outcomes, including mortality [8, 9] and acute exacerbation of chronic obstructive pulmonary disease [10]. ILAs frequently progress over time, with rates ranging from 20% over 2 years to 73% over 5 years [5, 11–13]. ILA progression is further associated with an increased risk of mortality [5, 12] and a decline in lung function [10, 12].

At present, when ILAs are initially suspected, the diagnostic approach typically involves additional prone chest CT imaging, particularly to differentiate basal subpleural abnormalities from dependent atelectasis [2, 14]. A previous study found that 7% of approximately 1000 health check-up subjects underwent additional prone CT due to parenchymal abnormalities, with a final early ILD detection rate of 0.3%, thereby recommending prone CT to distinguish early ILD from dependent densities [15]. However, if fibrotic features such as honeycombing or traction bronchiectasis are clearly present on the initial supine images, prone chest CT may not be necessary to confirm the presence of ILAs. In another study, prone CT did not improve honeycombing identification or diagnostic accuracy of usual interstitial pneumonia for both experienced radiologists and trainees, although less experienced trainees benefited in classifying usual interstitial pneumonia [16]. Optimal utilization of prone CT in suspected ILA cases can minimize unnecessary imaging, reduce radiation exposure and patient inconvenience, and streamline the diagnostic process. In this study, we aimed to evaluate whether supine chest CT alone is sufficient for diagnosing ILAs based on their types: fibrotic and non-fibrotic ILAs. We hypothesized that prone imaging would be redundant for diagnosing fibrotic ILAs.

## Materials and methods

This retrospective study was approved by the institutional review board of Seoul National University Hospital (IRB No. 2401-038-1501), and the requirement for informed consent was waived.

### Patient cohort

We first identified patients who underwent thin-section (slice thickness less than 2 mm) prone chest CT from January 2021 to July 2023. Then, we applied the following inclusion criteria: (1) patients who had a supine chest CT taken within the previous year; (2) the prior supine chest CT showed abnormalities suspected of ILAs or requiring exclusion of ILAs, leading to subsequent prone CT. Exclusions were made for patients with known ILD, clinical suspicion of ILD, follow-up studies of known ILAs, prone CT taken for other indications (e.g., dyspnea workup or follow-up for known pulmonary pathology such as pneumonia), and cases with poor image quality. A consensus assessment was performed by two board-certified radiologists (J.S. and S.H.Y., with 4 and 19 years of experience in thoracic CT interpretation, respectively) to determine the presence of ILAs and their subtypes. The validity of the reference labels was examined by comparing them with the majority interpretations of five multinational experts in thoracic radiology who were independent of the two radiologists. From this cohort of patients who underwent both supine and prone CT scans, we randomly selected 23 age- and sex-matched patients for each category—normal lung, non-fibrotic ILAs, and fibrotic ILAs—resulting in a total of 69 patients. CT scan protocol details are described in Table S1.

The necessary sample size to compare the areas under the receiver operating characteristic curve (AUCs) of supine CT alone and combined supine-prone CT was calculated using MedCalc version 22 [17]. Given the lack of relevant prior studies, we assumed an AUC of 0.75 for supine CT alone and an AUC of 0.95 for combined supine-prone CT, with a correlation between the two methods of 0.5, indicating moderate agreement. Setting the ratio of normal to ILA subjects as 1:1, the estimated sample size for each group was 23 patients. Based on our available sample, a difference in AUC of 0.2 would be detectable.

### Image interpretation

In session 1, five board-certified thoracic radiologists from Korea, Japan, and the United States (D.A.L., M.Y., J.H.C., K.J.C., and J.E.L. with 37, 24, 15, 14, and 10 years of experience in thoracic CT interpretation, respectively), who were blinded to patient information and CT interpretations, independently rated ILA suspicion scores on a 5-point scale using supine CT images. When they suspected the presence of an ILA, indicated by an ILA suspicion score of 3 points or more, they assigned a fibrosis score (1 to 5 points) and ILA extent (1–100%). After a 1-month washout period, matched supine-prone CT images were provided in session 2, replicating clinical situations where prone CT is performed in patients with suspected ILAs on supine CT. We classified each reader’s ILA suspicion and fibrosis scores into four diagnostic categories (normal, non-fibrotic ILAs, indeterminate-type ILAs, and fibrotic ILAs). ILA suspicion scores of 1 and 2 were regarded as normal, while scores of 3 to 5 were considered positive for ILAs. For positive ILA cases, fibrosis scores of 1 and 2 were classified as non-fibrotic ILAs, 3 as indeterminate-type ILAs, and 4–5 as fibrotic ILAs.

### Study outcomes

After dividing the study cohort into two groups, one comprising normal and non-fibrotic ILA patients and the other including normal and fibrotic ILA patients, we evaluated differences in diagnostic performance between session 1 (supine CT only) and session 2 (both supine and prone CT) according to ILA subtypes. Next, we evaluated inter-reader agreement on the diagnostic categories, which we reclassified into four categories based on the ILA suspicion scores and fibrosis scores. Lastly, regarding ILA extent, we evaluated intra-rater and inter-rater reliability and calculated the mean differences between the sessions for each ILA subtype.

### Statistical analysis

Statistical testing was performed by an expert biostatistician (M.J.J.) using SAS version 9.4 and R version 4.2.1. To compare patient demographics, the Kruskal–Wallis rank sum test was performed for continuous variables, and the Pearson chi-square test or Fisher exact test was used for categorical variables.

To assess potential differences in diagnostic performance between sessions across ILA subtypes, we compared the AUCs of ILA suspicion scores provided by the five readers between sessions. To pool the AUCs of multiple readers for each session and compare the AUCs of two sessions, the random-reader model of the Obuchowski–Rockette method for multi-reader multi-case studies [18] was used. The analysis was conducted using the R package MRMCaov [19].

Inter-reader agreement on the diagnostic categories was assessed by using the Fleiss kappa and Gwet AC1 for all five readers and the Cohen kappa and Gwet AC1 for pairwise assessments. The 95% confidence intervals and *p*-values for comparisons of the Fleiss kappa and Gwet AC1 between sessions were obtained from 1000 rounds of bootstrap replications.

Intra-rater reliability in the evaluation of ILA extent was assessed by calculating concordance correlation coefficients (CCCs) between sessions for each reader. The variance components approach was used to estimate the overall CCCs for five readers [20]. Inter-rater reliability was evaluated by calculating intraclass correlation coefficients (ICCs) across the five readers’ assessments of extent for each session and ILA type. Bootstrapping was performed 1000 times to obtain 95% confidence intervals and *p*-values for comparison. The mean differences between sessions were evaluated using a linear mixed model. Reader-specific cases classified as normal (with ILA suspicion score 1 or 2), the extent of which was not evaluated, were excluded from the analysis.

## Results

### Patient demographics

**Fig. 1:**
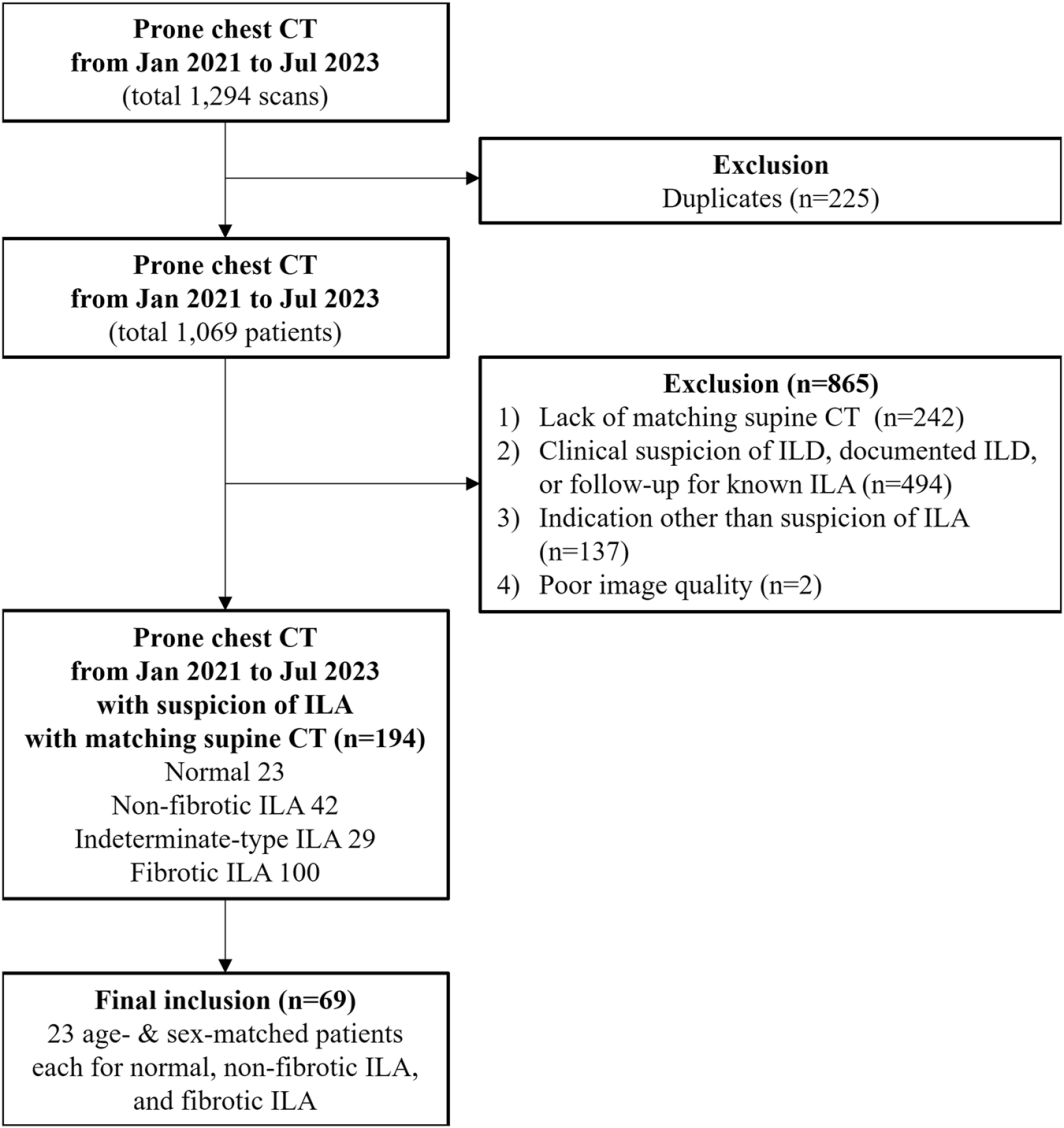
Flowchart of study population selection

From January 2021 to July 2023, a total of 1294 chest CT exams containing prone scans were identified. After removing duplicates, it was found that 1069 patients underwent prone chest CT during this period. Among them, 242 patients were excluded due to a lack of matching supine CT scans. Another 494 patients were excluded because the prone CT scans were taken with clinical suspicion of ILD, due to documented ILD, or for follow-up of known ILAs. Additionally, 137 patients were excluded due to prone CT taken for indications other than suspicion of ILAs, and 2 patients were excluded due to inadequate image quality, leaving 194 candidate subjects. These included 23 subjects with normal findings, 42 patients with non-fibrotic ILAs, 29 patients with indeterminate-type ILAs, and 100 patients with fibrotic ILAs. We randomly selected 23 patients each from the non-fibrotic and fibrotic ILA groups, ensuring that their age and sex distributions matched those of the normal group. Finally, 69 patients, including 23 each from the normal, non-fibrotic ILA, and fibrotic ILA groups, were evaluated by five thoracic radiologists. The subject selection flowchart is depicted in Fig. 1. Table 1 shows the demographic data of these 69 patients. Thirty-six were women and 33 were men, with a median age of 69 years. No significant differences in the age and sex distribution were observed across the diagnostic groups. The mean dose-length product for supine CT imaging was 100.6 mGy*cm, and 63.6 mGy*cm for prone CT imaging (Table S1).

**Table 1:** Patient demographics

Characteristics	Overall	Normal	Non-fibrotic ILA	Fibrotic ILA	*p*-value^c^
No. of participants	69	23	23	23	
Women	36 (52.2)	12 (52.2)	12 (52.2)	12 (52.2)	> 0.99
Age, mean^a^	67.2 ± 7.2	65.9 ± 8.3	67.1 ± 7.8	68.6 ± 5.1	
Age, median^b^	69 (63, 72)	69 (61, 71)	68 (63, 75)	69 (67, 72)	0.64
Age group					0.92
40–49 years	2 (2.9)	1 (4.3)	1 (4.3)	0 (0.0)	
50–59 years	6 (8.7)	2 (8.7)	1 (4.3)	3 (13.0)	
60–69 years	31 (44.9)	9 (39.1)	10 (43.5)	12 (52.2)	
70–79 years	30 (43.5)	10 (43.5)	10 (43.5)	10 (43.5)	

Data are numbers of patients with the proportion in parentheses unless otherwise indicated

^a^ Data are means ± SDs

^b^ Data in parentheses are the first quartile and third quartile

^c^ Sex distribution was assessed using the Pearson chi-square test, age was evaluated using the Kruskal–Wallis rank sum test, and age group was analyzed using the Fisher exact test

### Diagnostic performance according to ILA subtypes

**Fig. 2:**
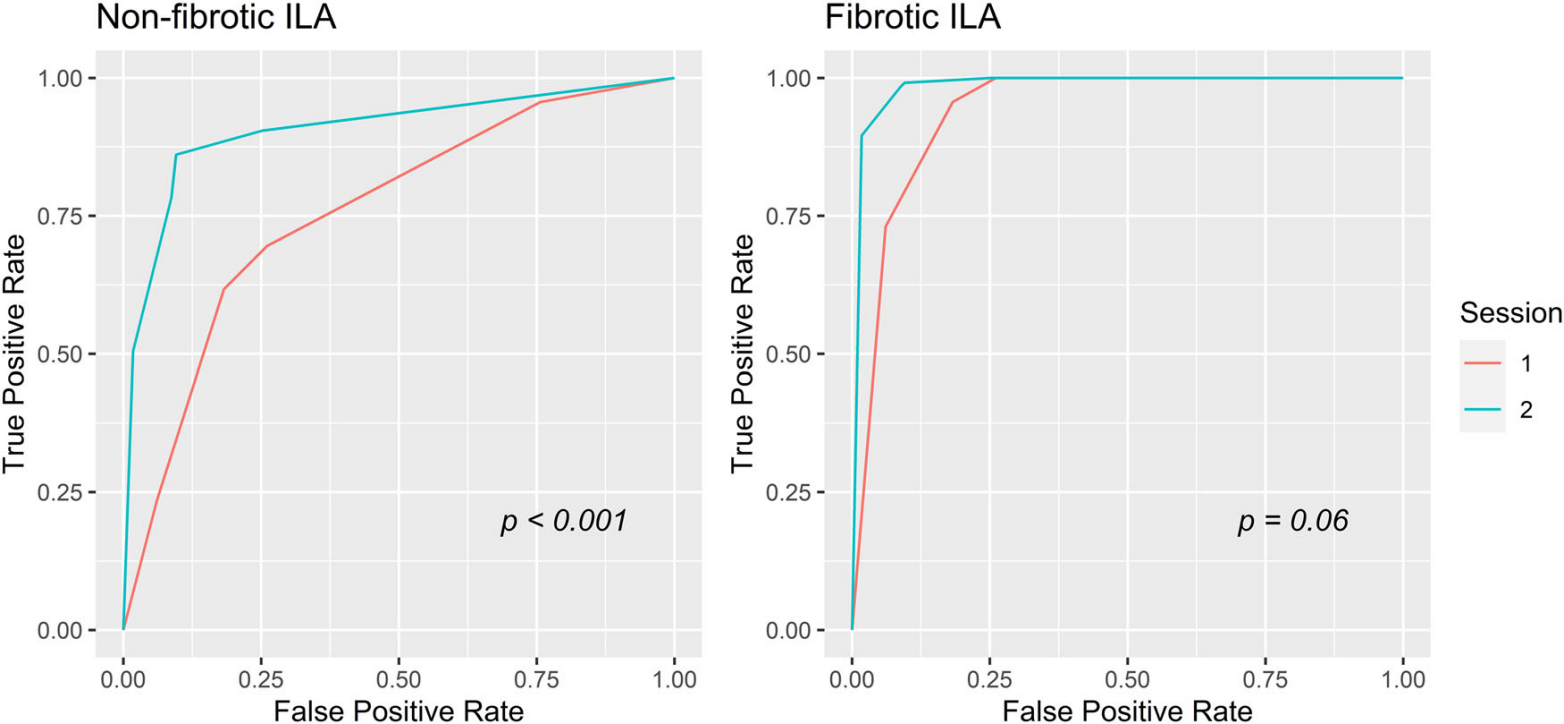
Pooled receiver operating characteristic curves of all five readers’ ILA suspicion scores for diagnosing ILA in non-fibrotic and fibrotic ILA subgroups

For all cases, the reference standard labels of ILA status and type, determined by consensus of two radiologists, matched the majority results of at least three of the five readers. The pooled AUCs for discriminating non-fibrotic ILAs from normal findings were 0.76 in session 1 and significantly improved to 0.92 in session 2 (*p* < 0.001). For discriminating fibrotic ILAs from normal findings, the pooled AUCs were 0.94 in session 1 and 0.99 in session 2, showing no significant difference (*p* = 0.06). The reader-specific AUCs and pooled AUCs from the random-reader model for each session by ILA subtypes are shown in Table 2, and the receiver operating characteristic curves are illustrated in Fig. 2 (pooled) and Fig. S1 (reader-specific).

**Table 2:** The reader-specific AUCs and pooled AUCs from the random-reader model for each session and ILA type

		Non-fibrotic		Fibrotic	
Reader	Session	AUC (95% CI)	*p-*value	AUC (95% CI)	*p-*value
1	Supine CT only	0.79 (0.66, 0.92)		0.88 (0.79, 0.98)	
	Supine and prone CT	0.94 (0.88, 1.00)		1.00 (0.99, 1.00)	
	Difference	−0.15 (−0.26, −0.04)	0.006	−0.12 (−0.21, −0.02)	0.02
2	Supine CT only	0.73 (0.59, 0.87)		0.95 (0.90, 1.00)	
	Supine and prone CT	0.87 (0.78, 0.97)		1.00 (1.00, 1.00)	
	Difference	−0.14 (−0.25, −0.03)	0.01	−0.05 (−0.10, 0.00)	0.05
3	Supine CT only	0.73 (0.6, 0.86)		0.95 (0.91, 1.00)	
	Supine and prone CT	0.91 (0.83, 1.00)		0.97 (0.93, 1.00)	
	Difference	−0.18 (−0.3, −0.06)	0.004	−0.02 (−0.07, 0.03)	0.49
4	Supine CT only	0.72 (0.58, 0.87)		0.92 (0.84, 1.00)	
	Supine and prone CT	0.93 (0.86, 1.00)		0.97 (0.92, 1.00)	
	Difference	−0.21 (−0.35, −0.08)	0.002	−0.05 (−0.14, 0.04)	0.27
5	Supine CT only	0.80 (0.68, 0.92)		0.99 (0.97, 1.00)	
	Supine and prone CT	0.92 (0.84, 1.00)		1.00 (0.99, 1.00)	
	Difference	−0.12 (−0.25, 0.00)	0.055	−0.01 (−0.03, 0.01)	0.32
Pooled	Supine CT only	0.76 (0.67, 0.84)		0.94 (0.89, 0.99)	
	Supine and prone CT	0.92 (0.85, 0.98)		0.99 (0.97, 1.00)	
	Difference	−0.16 (−0.22, −0.11)	< 0.001	−0.05 (−0.10, 0.00)	0.06

*CI* confidence interval

**Fig. 3:**
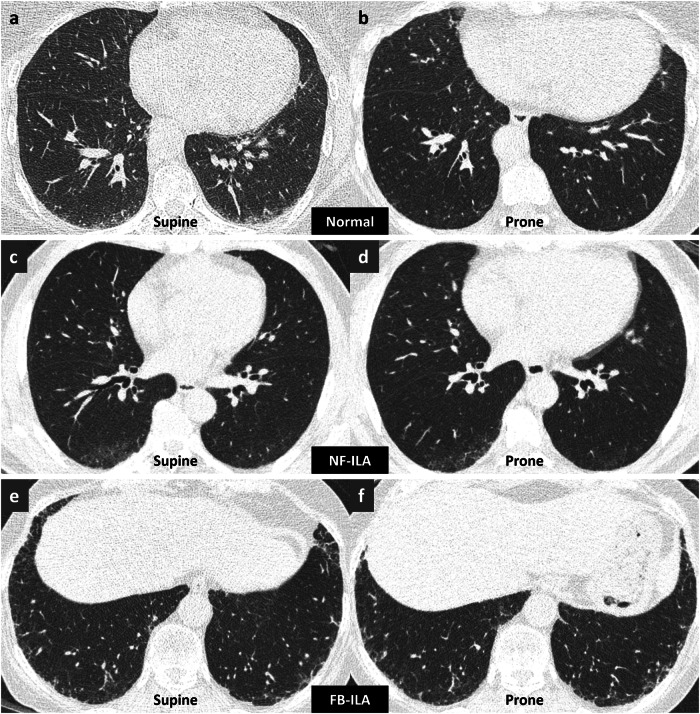
Chest CT images of exemplary cases of a normal subject (**a**, **b**), a patient with non-fibrotic ILA (**c**, **d**), and a patient with fibrotic ILA (**e**, **f**). Subtle ground-glass opacities in the posterior subpleural portion are seen on (**a**) supine CT and were initially evaluated as positive for ILA by the majority of readers using supine CT alone. The lesions disappeared on (**b**) prone CT, indicating dependent atelectasis. Subtle ground-glass opacities in the posterior subpleural portion are seen on (**c**) supine CT and (**d**) persist in prone CT, indicating non-fibrotic ILA. Mild reticular opacities with traction bronchiolectasis in the subpleural portion are noted in both (**e**) supine and (**f**) prone CTs. The majority of readers identified fibrotic ILAs in supine CT alone and supine-prone CTs

Figure 3 illustrates exemplary cases of normal, non-fibrotic, and fibrotic ILA subjects. In certain instances of normal (Fig. 3a, b) and non-fibrotic ILAs (Fig. 3c, d), the majority of readers revised their assessment of ILA presence upon addition of prone CT, whereas such changes were not observed in fibrotic ILA subjects (Fig. 3e, f).

### Reader agreement on diagnostic category

**Fig. 4:**
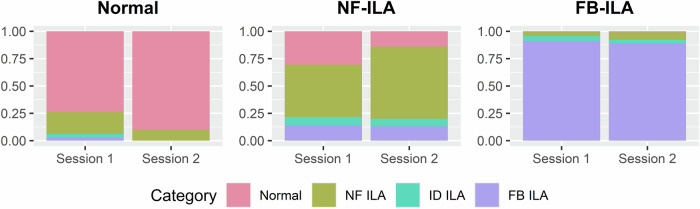
Distributions of readers’ diagnostic categories. NF ILA, non-fibrotic ILA; ID ILA, indeterminate-type ILA; FB ILA, fibrotic ILA

The overall and reader-specific distributions of diagnostic categories based on ILA suspicion scores and fibrosis scores are illustrated in Fig. 4 and S2. In session 1, readers correctly classified normal findings in 74% of cases, non-fibrotic ILAs in 48%, and fibrotic ILAs in 91% of cases. In session 2, the matching rates were 90% for normal findings, 66% for non-fibrotic ILAs, and 89% for fibrotic ILAs.

The overall Fleiss kappa and Gwet AC1 values for session 1 in the normal and non-fibrotic ILA subgroups were 0.25 and 0.44, respectively. In session 2, these values significantly increased to 0.51 and 0.65 (*p* = 0.004 for both). However, there was no significant improvement in the overall Fleiss kappa and Gwet AC1 in the normal and fibrotic ILA subgroup; these values were 0.63 and 0.70 in session 1 and 0.72 and 0.80 in session 2, respectively (*p* = 0.082 for the Fleiss kappa and *p* = 0.064 for the Gwet AC1).

The pairwise agreement of all reader pairs in terms of the Cohen kappa coefficient ranged from 0.11 to 0.43 in session 1 and from 0.28 to 0.73 in session 2 for the normal and non-fibrotic ILA subgroups. For the normal and fibrotic ILA subgroups, the Cohen’s kappa of all reader pairs ranged from 0.53 to 0.74 in session 1 and from 0.61 to 0.88 in session 2 (Table S2).

### Reliability of extent evaluation

The intra-reader CCC was 0.69 (95% CI: 0.54–0.79) for fibrotic ILAs and 0.41 (95% CI: 0.21–0.57) for non-fibrotic ILAs when excluding missing values. While the numerical value of intra-reader reliability appeared to be higher for fibrotic ILAs than for non-fibrotic ILAs, statistical significance was not demonstrated (*p* = 0.14).

The assessment of extent in session 1 revealed higher inter-reader reliability for fibrotic ILAs (ICC: 0.56, 95% CI: 0.30–0.70) than for non-fibrotic ILAs (ICC: 0.22, 95% CI: 0.04–0.31; *p* = 0.02). However, in session 2, which added prone images for evaluation, there was no significant difference in inter-reader reliability between ILA types (ICC: 0.59 [95% CI: 0.21–0.67] for fibrotic ILAs; ICC: 0.31 [95% CI: 0.07–0.46] for non-fibrotic ILAs; *p* = 0.12). The inclusion of prone images in session 2 did not lead to a significant improvement in the inter-reader reliability for either ILA type (session 1 versus session 2, *p* = 0.73 for fibrotic ILAs and *p* = 0.22 for non-fibrotic ILAs) (Table 3).

**Table 3:** Intra-rater concordance correlation coefficients between sessions and intraclass correlation coefficients among five readers for each session and ILA type regarding the extent evaluation

	Intra-rater CCC	Inter-rater ICC		
		Supine CT only	Supine and prone CT	*p*-value^b^
Fibrotic ILA	0.69 (0.54, 0.79)	0.56 (0.30, 0.70)	0.59 (0.21, 0.67)	0.73
Non-fibrotic ILA	0.41 (0.21, 0.57)	0.22 (0.04, 0.41)	0.31 (0.07, 0.46)	0.22
*p*-value^a^	0.13	0.02	0.12	

Missing values were excluded during the analysis. Data in parentheses are 95% confidence intervals

^a^ *p*-values comparing fibrotic and non-fibrotic ILAs

^b^ *p*-values comparing ICCs of session 1 (with supine CT only) and session 2 (with both supine and prone CT)

The mean ILA extent decreased from 6.7 to 5.4% for non-fibrotic ILAs from session 1 to session 2, with a mean difference of 1.3 percentage points (*p* < 0.001). For fibrotic ILAs, the mean extent decreased from 10.0% in session 1 to 8.1% in session 2, with a mean difference of 1.9 percentage points (*p* < 0.001).

## Discussion

The study showed that the value of adding prone CT to supine CT varied between fibrotic and non-fibrotic ILAs. Adding prone CT significantly improved performance in differentiating non-fibrotic ILAs from normal cases (pooled AUC 0.76 to 0.92, *p* < 0.001), but not for distinguishing fibrotic ILAs from normal cases (pooled AUC 0.94 and 0.99, *p* = 0.06), indicating that fibrotic features can be adequately captured on supine CT alone. The inter-reader agreement for diagnostic categories improved when prone CT was added for non-fibrotic ILAs (Fleiss kappa, 0.25 versus 0.51, *p* = 0.004), while no difference was noted for fibrotic ILAs (Fleiss kappa, 0.63 versus 0.72, *p* = 0.08). Readers tended to overestimate the extent by 1.3 percentage points for non-fibrotic ILAs and 1.9 percentage points for fibrotic ILAs on supine CT alone, both corresponding to 24% of the mean extent evaluated on both supine and prone CTs.

Several studies have reported that the risk of progression and mortality differs according to the type of ILA. Jin et al reported that fibrotic ILAs progressed in 37% of cases with no improvement at the 2-year follow-up, whereas non-fibrotic ILAs progressed in 11% and showed improvement in approximately half of the cases [11]. Putman et al reported that ILAs with definite fibrosis had eight times higher odds of progression on imaging over 5 years and a 70% higher risk of death compared to ILAs without fibrosis [5]. These findings underscore the importance of accurately diagnosing fibrotic ILAs, given their closer association with progression and increased mortality. Most previous studies on ILAs relied solely on diagnoses from supine CT, which might be criticized for potential mislabeling dependent atelectasis as ILAs. Our results provide evidence that even without confirming fibrotic ILAs on prone CT, the current knowledge from the literature remains valid, at least for fibrotic ILAs.

Previous studies have reported various levels of inter-reader agreement for ILA subtypes. Park et al reported a kappa value of 0.47 for ILA subtypes [21], while Chae et al reported a kappa value of 0.76 [22]. The major difference between the two studies was the exclusion criteria: Park et al did not exclude cases with dependent or passive atelectasis, whereas Chae et al excluded those cases. In our study, the kappa values for ILA subtypes were 0.25 for the normal/non-fibrotic ILA subgroup and 0.63 for the normal/fibrotic ILA subgroup with supine CT alone. These values improved to 0.51 and 0.72, respectively, with combined supine-prone CT. When evaluated with only supine CT, our results were comparable to those of Park et al, showing moderate agreement. With combined supine-prone CT, our results were comparable to those of Chae et al, showing good agreement. This may suggest that the need to differentiate dependent atelectasis impacted inter-reader agreement, especially when a non-fibrotic ILA was suspected.

The intra-rater reliability for ILA extent was not very high for either ILA type, which was likely influenced by the tendency to overestimate ILA extent using supine CT alone. Dependent atelectasis and the associated decrease in lung volume in the subpleural area when supine positioning likely contributed to some overestimation. The slightly better inter-reader reliability for fibrotic ILAs than for non-fibrotic ILAs with supine CT alone further suggests that dependent atelectasis, which can mimic non-fibrotic ILAs [1], may have contributed to extent overestimation. The general lack of intra- and inter-reader consistency in determining ILA extent may also have contributed to the overall low reliability. Interestingly, adding prone CT did not significantly improve inter-rater reliability for either ILA type, indicating that prone CT may not play a significant role in improving reader agreement in this regard. The main consideration when using supine CT alone should be the slight overestimation of extent.

Several limitations should be acknowledged in this study. The retrospective nature, small sample size, and inclusion criteria requiring prior supine CT may introduce selection bias and limit the generalizability of the results. In addition, the case-control design, which artificially composes normal and ILA cases, is not ideal for diagnostic studies or AUC calculations, as it fails to reflect the actual prevalence and distribution of ILA subtypes. Future studies should utilize larger cohorts and prospective designs where all suspected ILAs undergo both supine and prone CT to enhance the generalizability of our findings. Moreover, the subjective nature of determining ILA types made it difficult to establish a gold standard. Nonetheless, the current standard established by two readers aligned with the majority consensus of the five other independent readers. Finally, while this study included only expert thoracic radiologists, future research could incorporate readers with varying experience levels to investigate how reader experience affects the necessity of prone CT in diagnosing ILAs.

In conclusion, our study provides evidence supporting the use of supine chest CT imaging alone for diagnosing fibrotic ILAs, while the diagnosis of non-fibrotic ILAs benefited from adding prone CT. Supine CT alone led to a slight overestimation of ILA extent regardless of ILA type. Omitting prone CT for fibrotic ILAs could potentially reduce unnecessary imaging time and the radiation exposure associated with prone chest CT, thereby streamlining patient management.

## Supplementary information

ELECTRONIC SUPPLEMENTARY MATERIAL

## Abbreviations

AUC: Area under the receiver operating characteristic curve
CCC: Concordance correlation coefficient
CI: Confidence interval
ICC: Intraclass correlation coefficient
ILA: Interstitial lung abnormality
ILD: Interstitial lung disease
