# Supplementary material for: Optimizing prone CT use for suspected interstitial lung abnormalities
Source: Eur Radiol. 2024 Dec 18;35(6):3021–9. doi: 10.1007/s00330-024-11259-5 (PMC12081487; doi:10.1007/s00330-024-11259-5)
Supplement: Supplementary file 1 — ELECTRONIC SUPPLEMENTARY MATERIAL [file 330_2024_11259_MOESM1_ESM.pdf]

Optimizing Prone CT Use for Suspected Interstitial Lung Abnormalities

ELECTRONIC SUPPLEMENTARY MATERIAL

Eur Radiol (2024) Song J, Chae KJ, Lee JE, et al.

Figure S1. Receiver operating characteristic curves of the reader-specific ILA suspicion score for diagnosing ILA status in non-fibrotic and fibrotic ILA subgroups

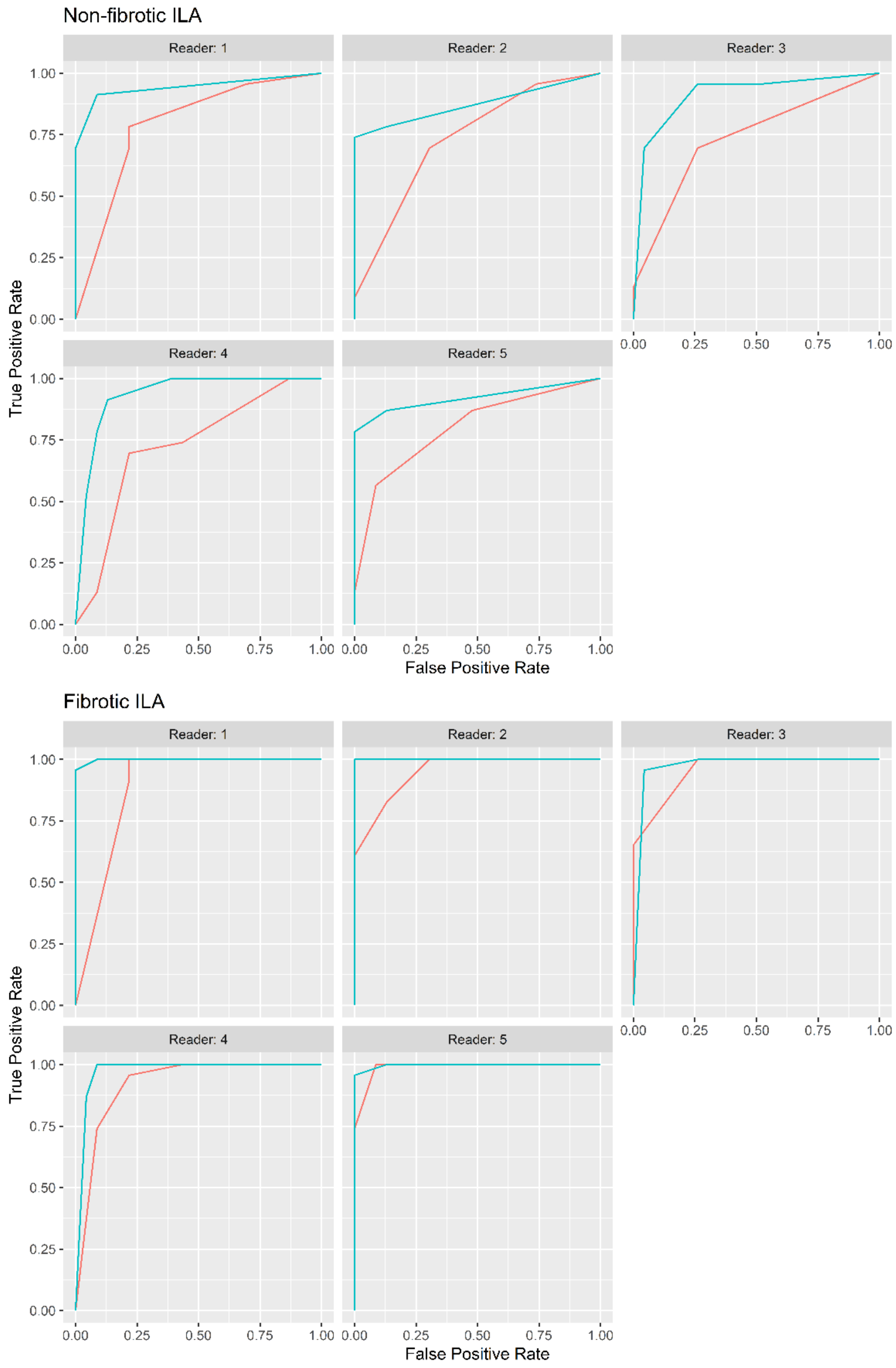

Figure S2. Distributions of overall and reader-specific diagnostic categories

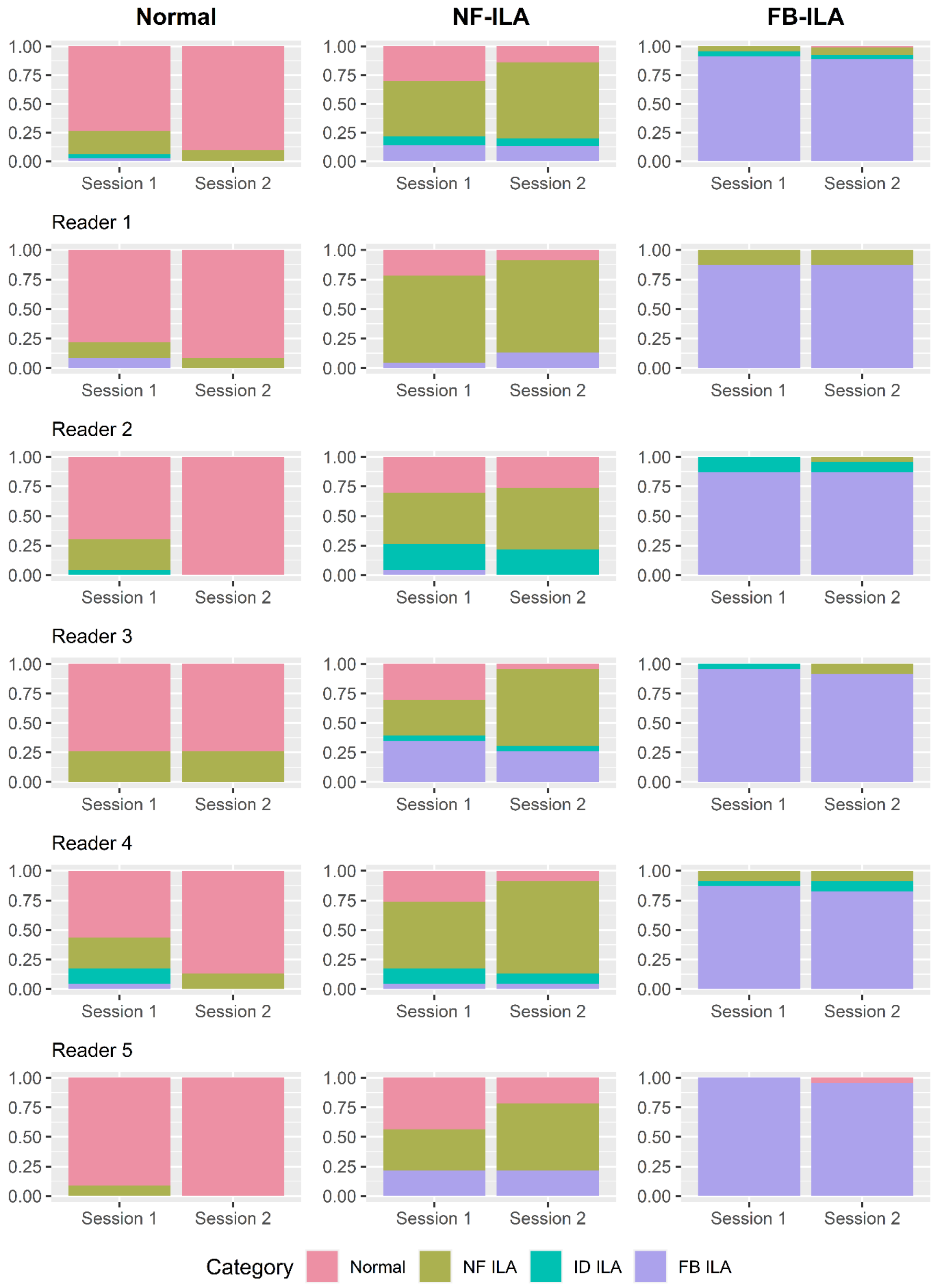

Eur Radiol (2024) Song J, Chae KJ, Lee JE, et al.

Supplementary Tables

Table S1. Summary of CT scanning protocols

	**Supine CT**	**Prone CT**
**kVp**	120 (n=64); 100 (n=4); 70 (n=1)	120 (n=65); 100 (n=4)
**Radiation dose**†	$CTDI_{vol}$: 2.5 ± 1.2 mGy<br>DLP: 100.6 ± 41.8 mGy*cm	$CTDI_{vol}$: 1.5 ± 0.5 mGy<br>DLP: 63.6 ± 20.6 mGy*cm
**Slice thickness (mm)**	0.75 (n=1);<br>1 (n=65);<br>1.25 (n=2);<br>2 (n=1)	1 (n=64);<br>1.25 (n=5)
**Field of view (mm)**	285-384	291-427
**Kernel**	YC (n=55); LUNG (n=3); B60f (n=3); B70f (n=2); Br59d, Bv40d, FC07, I50f, I70f, YA (n=1)	YC (n=59); Br59d (n=4); STANDARD (n=4); I70f, LUNG (n=1)
**Machine & Manufacturers**	**Philips**<br>Ingenuity CT (n=47);<br>IQon-Spectral CT (n=6);<br>iCT 256 (n=2);<br>Brilliance 64 (n=1)<br>**Siemens**<br>Somatom Definition Flash (n=4);<br>Somatom Force (n=2);<br>Somatom Definition (n=2);<br>Somatom Definition Edge (n=1)<br>**GE** Revolution CT (n=3)<br>**Toshiba** Aquilion ONE (n=1)	**Philips**<br>Ingenuity CT (n=46);<br>IQon-Spectral CT (n=5);<br>iCT 256 (n=7);<br>Brilliance 64 (n=1)<br>**Siemens**<br>Somatom Force (n=4);<br>Somatom Definition Flash (n=1)<br>**GE**<br>Revolution Apex (n=4);<br>Discovery CT750 HD (n=1)

† Data are means ± SDs

$CTDI_{vol}$ = volume computed tomography dose index, DLP = dose-length product

Table S2. Pairwise inter-reader agreement of diagnostic categories according to ILA types and sessions

Type of ILA	Session	Reader A	Reader B	Cohen kappa (95% CI)	Gwet AC1 (95% CI)
Non-fibrotic	Supine CT only	1	2	0.31 (0.11, 0.51)	0.49 (0.30, 0.67)
			3	0.32 (0.11, 0.52)	0.48 (0.30, 0.67)
			4	0.25 (0.05, 0.46)	0.43 (0.24, 0.62)
			5	0.34 (0.13, 0.55)	0.49 (0.29, 0.70)
		2	3	0.11 (-0.08, 0.29)	0.29 (0.09, 0.48)
			4	0.24 (0.04, 0.44)	0.39 (0.20, 0.59)
			5	0.18 (-0.01, 0.37)	0.41 (0.22, 0.6)
		3	4	0.27 (0.08, 0.46)	0.39 (0.20, 0.58)
			5	0.43 (0.21, 0.64)	0.60 (0.43, 0.77)
		4	5	0.17 (-0.01, 0.34)	0.35 (0.16, 0.54)
	Supine & prone CT	1	2	0.51 (0.33, 0.69)	0.65 (0.49, 0.82)
			3	0.60 (0.40, 0.80)	0.70 (0.55, 0.86)
			4	0.73 (0.56, 0.89)	0.81 (0.68, 0.94)
			5	0.58 (0.39, 0.77)	0.67 (0.49, 0.85)
		2	3	0.28 (0.11, 0.44)	0.43 (0.24, 0.62)
			4	0.47 (0.29, 0.65)	0.63 (0.46, 0.80)
			5	0.52 (0.34, 0.70)	0.68 (0.52, 0.85)
		3	4	0.46 (0.26, 0.65)	0.59 (0.42, 0.77)
			5	0.51 (0.31, 0.71)	0.62 (0.45, 0.79)
		4	5	0.55 (0.37, 0.73)	0.68 (0.52, 0.84)
Fibrotic	Supine CT only	1	2	0.59 (0.42, 0.77)	0.67 (0.50, 0.84)
			3	0.61 (0.42, 0.79)	0.70 (0.54, 0.86)
			4	0.53 (0.35, 0.71)	0.61 (0.44, 0.79)
			5	0.74 (0.57, 0.90)	0.79 (0.64, 0.94)
		2	3	0.56 (0.39, 0.74)	0.64 (0.47, 0.81)
			4	0.64 (0.48, 0.81)	0.69 (0.53, 0.86)
			5	0.72 (0.56, 0.88)	0.78 (0.64, 0.92)
		3	4	0.57 (0.39, 0.74)	0.64 (0.47, 0.81)
			5	0.74 (0.58, 0.9)	0.81 (0.68, 0.94)
		4	5	0.59 (0.43, 0.75)	0.67 (0.50, 0.84)
	Supine & prone CT	1	2	0.74 (0.58, 0.89)	0.81 (0.68, 0.94)
			3	0.61 (0.43, 0.79)	0.66 (0.47, 0.84)
			4	0.86 (0.73, 0.99)	0.89 (0.79, 0.99)
			5	0.80 (0.66, 0.95)	0.85 (0.72, 0.98)
		2	3	0.65 (0.48, 0.81)	0.73 (0.57, 0.88)
			4	0.71 (0.55, 0.87)	0.78 (0.64, 0.92)
			5	0.88 (0.75, 1.00)	0.92 (0.83, 1.00)
		3	4	0.66 (0.49, 0.82)	0.72 (0.57, 0.88)
			5	0.67 (0.50, 0.83)	0.73 (0.56, 0.89)
		4	5	0.70 (0.53, 0.86)	0.79 (0.65, 0.92)
